# A Diagnostic Survey of Aborted Equine Fetuses and Stillborn Premature Foals in Denmark

**DOI:** 10.3389/fvets.2021.740621

**Published:** 2021-11-10

**Authors:** Jørgen Steen Agerholm, Eva-Maria Klas, Peter Damborg, Nicole Borel, Hanne Gervi Pedersen, Mette Christoffersen

**Affiliations:** ^1^Department of Veterinary Clinical Sciences, University of Copenhagen, Høje Taastrup, Denmark; ^2^Department of Molecular Biology, LABOKLIN GmbH & Co. KG, Bad Kissingen, Germany; ^3^Department of Veterinary and Animal Sciences, University of Copenhagen, Frederiksberg, Denmark; ^4^Institute of Veterinary Pathology, Vetsuisse Faculty University Zurich, Zurich, Switzerland

**Keywords:** *Acinetobacter hydrophila*, *Arthrobacter gandavensis*, *Chlamydia*, *Enterococcus casseliflavus*, equine herpesvirus, *Pseudomonas fluorescens*, *Staphylococcus vitulinus*, *Streptococcus equi*

## Abstract

**Background:** Loss of pregnancy in mares can have many different causes, including both infectious and non-infectious conditions. Extrapolation of findings from other studies is often uncertain as the significance of each cause varies across regions. Causes of pregnancy loss in mares have never been thoroughly studied in Denmark, so a prospective cross-sectional cohort study targeting the entire Danish population of pregnant mares was performed over a period of 13 months to obtain knowledge of the significance of individual causes. Fifty aborted or prematurely delivered stillborn fetuses were submitted for necropsy and examined by a panel of diagnostic laboratory methods.

**Results:** Overall, a cause of fetal loss was established for 72% of the examined cases. Most cases (62%) were lost due to a non-infectious cause, of which obstruction of the feto-placental blood circulation due to severe torsion of the umbilical cord was most prevalent. Pregnancy loss due to a variety of opportunistic bacteria, including bacteria not previously associated with abortion in mares, accounted for 12%, while equid alphaherpesvirus (EHV) type 1 was the cause of pregnancy loss in 8% of the cases. EHV type 4 and *Chlamydiaceae* species were identified in some cases, but not regarded as the cause of fetal loss.

**Conclusion:** Umbilical cord torsion was found to be the most prevalent cause of fetal loss in Danish mares, while infectious causes such as EHV type 1 and streptococci only accounted for a minor proportion of the losses. The study highlights the need for defined criteria for establishing an abortion diagnosis in mares, particularly in relation to EHV types 1 and 4.

## Introduction

Pregnancy loss in mares may occur at any stage of gestation from fertilization to parturition. Analysis of breeding data indicates that 25–40% of mares that are bred fail to produce a viable offspring (1). However, these data include not only embryonic and fetal losses, but also mares that fail to conceive. More accurate data on fetal loss have been obtained by analyzing breeding data of intensively managed Thoroughbred mares in the UK and Ireland, where fetal losses from gestation day (GD) 70–300, 301–315, and stillbirth/perinatal death accounted for 4, 0.3, and 1.4%, respectively (2). Platt (3) also identified a pregnancy loss rate of 12.8% in English Thoroughbred mares that were confirmed pregnant by rectal palpation around GD 42 but either aborted or delivered a stillborn foal.

Diagnostic studies of aborted, premature, and stillborn foals have revealed a wide range of causes (4–13). However, the results of these studies cannot be directly compared, since different study designs, methods, and diagnostic criteria, were used by different researchers. Furthermore, since these studies were conducted in different geographical regions, the results could have been influenced by management procedures, local prevalence of pathogens, vaccination programs, etc. Diagnostic surveys of well-defined geographic regions are therefore needed to obtain in-depth knowledge about the regional or local causes of pregnancy loss in mares.

Knowledge about the causes of equine abortion and prematurely delivered stillborn foals in Denmark is currently extremely scarce. Systematic diagnostic surveys have never been performed and cases are rarely submitted for necropsy-based diagnostics, primarily due to the costs associated with laboratory investigations. In this study, we report the results of a diagnostic survey of aborted equine fetuses and prematurely delivered stillborn foals over the course of 1 year in Denmark.

## Materials and Methods

The study was performed as a prospective cross-sectional cohort study targeting the entire Danish population of pregnant mares over a period of 13 months (November 1, 2018 to November 31, 2019). The horse population in Denmark comprised around 175,000 animals in 2019 and around 6,700 foals were born that year.

Horse owners whose mares aborted (expulsion of the conceptus ≤ 300 days of gestation) or delivered a stillborn foal prematurely (expulsion >300 and ≤ 315 days) were invited to submit the fetus including fetal membranes for a free diagnostic examination. Information regarding this service was shared through social media, including a Facebook group for Danish equine veterinarians and through direct contact with breeding centers and stud farms, which disseminated the information through their networks.

Fetuses and fetal membranes (i.e., allantochorion, amnion, and umbilical cord) were submitted to the Department of Veterinary Clinical Sciences, University of Copenhagen by courier or were delivered directly by the owner. A questionnaire was emailed to the owners with questions about the mare's breed, age, parity, previous fertility, breeding method, breeding date(s), date of delivery, vaccination status for equid alphaherpesvirus types 1 and 4 (EHV-1 and EHV-4, respectively), and clinical course of delivery.

For inclusion in the study, the following criteria had to be met: (1) gestational age of ≤ 315 days, (2) the material should be appropriate for examination, e.g., not macerated or frozen, and (3) the owner should provide data on the mare and give written consent to participate in the study. Eleven submissions were retrospectively excluded as they did not meet one of these requirements, in most cases a gestational age of >315 days (Supplementary Table 1).

Gestational age was calculated based on the breeding date. If the mare was hand bred or pasture bred for several days, the breeding date was set as the mid-point of the breeding period. Abortion of twins counted as a single case.

### Necropsy

Straw and dirt were initially rinsed from the fetus using low pressure tap water, before it was weighed, measured (crown-rump length), and fetal developmental characteristics and gender were recorded. The fetus was inspected for external lesions and then opened in the ventral midline. Specimens were taken for polymerase chain reaction (PCR) analysis and microbial culture before further handling of the fetus using sterile equipment and new examination gloves and placed in separate plastic containers. The necropsy was then completed and tissues sampled for histopathology.

The fetal membranes were spread out on the examination table to allow for thorough inspection. If severely contaminated by dirt and straw, they were initially rinsed by gentle immersion in a bucket of cold running tap water. Specimens for histopathology were sampled from the allantochorion of each horn (midpart) and the body near the cervical star. A specimen for microbial culture was taken from the body near the cervical star and placed in a plastic container. If gross lesions were observed, sampling was modified to also include these areas. The umbilical cord was inspected, the length measured, and representative specimens were collected for histopathology if lesions were present.

### Histopathology

Specimens of the lung, heart, liver, spleen, kidney, adrenal gland, thymus, skeletal muscle (*Musculus semitendinosus*), cerebrum, brain stem, cerebellum, umbilical cord, and allantochorion were placed in 10% neutral buffered formalin and fixed for around 3 weeks before processing. The tissues were then trimmed, processed by standard procedures, embedded in paraffin, sectioned at ~2 μm, and stained by hematoxylin and eosin (HE).

### Microbial Culture

Specimens of lung, liver, and allantochorion, as well as a sample of stomach content were collected at necropsy. If gross lesions indicative of inflammation were observed at necropsy, the samples were submitted for microbial culture after storage overnight at 5°C. Otherwise, the specimens were stored at −20°C until the results of the histopathology became available, and if inflammation was diagnosed at this stage, the specimens were thawed and cultured. Samples were plated in parallel on 5% calf blood agar and Sabouraud agar for bacteriological and mycological culture, respectively. Stomach content and a chorionic surface swab of allantochorionic samples were plated directly on these agars, while lung and liver samples were scalded for 5 s before plating a sample of material from below an incision. Both agar plates were incubated aerobically at 37°C. Subcultures of each morphologically distinct colony type observed at 24 and 48 h were examined by matrix-assisted laser desorption/ionization time of flight (MALDI-TOF) mass spectrometry using *Escherichia coli* ATCC 8739 as a reference strain and Saramis™ 3.5 for spectra interpretation.

### PCR Analysis for EHV-1, EHV- 4, Equine Arteritis Virus, and *Leptospira* spp.

Specimens of the lung, liver, and kidney were sampled at necropsy and shipped overnight by a courier service to LABOKLIN GmBH and Co. KG, Bad Kissingen, Germany for real-time PCR analysis. The tissues were either shipped on the day of necropsy or stored at −20 °C for a number of days (e.g., over the weekend) before shipping. The samples were tested for EHV-1 and EHV-4, equine arteritis virus (EAV) and *Leptospira interrogans* serovars Australis, Autumnalis, Bataviae, Bratislava, Canicola, Copenhageni, Djasiman, Hardjo, Icterohaemorrhagiae, Kennewicki, Mankarso, Pomona, Pyrogenes, and Wolffi.

Upon arrival, samples were barcoded for automatic processing. Lung and liver samples were pooled and analyzed together, while the kidney sample was processed separately. For DNA/RNA isolation, 10–20 mg of each tissue was placed in 50 μL proteinase K and 500 μL of tissue lysis buffer in green bead tubes, subjected to oscillation by the MagNA Lyser (Roche Diagnostics GmbH, Mannheim, Germany) for 40 s at 4,100 *g* and then incubated at 65°C for 1 h. Supernatant (200 μL) was automatically pipetted by a Hamilton Microlab STARlet (Hamilton Germany GmbH – Robotics, Gräfelfing, Germany) into a MagNA Pure 96 Processing Cartridge (Roche Diagnostics GmbH). DNA/RNA isolation was performed by the MagNA Pure with the MagNA Pure 96 DNA Kit and the Viral NA small volume kit (Roche Diagnostics GmbH). To control that the extraction of DNA/RNA was successful, an extraction control using the DNA or RNA Process Control (Detection) Kit (Roche Diagnostics GmbH) was run with every PCR.

To prepare the Master mixes for *Leptospira* spp., EHV-1, and EHV-4, the DNA Process Control Detection Kit (Roche Diagnostics GmbH) was used, while the RNA Process Control Detection Kit (Roche Diagnostics GmbH) was used for EAV. The Master mixes containing the primers and the enzymes were automatically pipetted and mixed with the DNA/RNA by another Hamilton Microlab STARlet (Hamilton Germany GmbH) in a separate clean room. Positive and negative controls were included with every PCR, which was performed using a LightCycler® 96 (Roche Diagnostics GmbH).

The PCRs for EHV-1 and EHV-4 targeted the *glycoprotein B* gene according to Diallo et al. (14, 15). When EHV-1 DNA was detected, an additional PCR targeting the *DNA polymerase* gene was performed according to Nugent et al. (16) to differentiate between neuropathogenic (D752) and non-neuropathogenic (N752) variants of EHV-1. The PCR for EAV targeted the *ORF7* gene and was designed based on the study by Lu et al. (17), while the PCR for *Leptospira* spp. was performed according to Stoddard et al. (18).

### PCR for *Chlamydiaceae* spp.

A 20 μm section of formalin-fixed and paraffin-embedded allantochorionic specimen from each case was cut and stored in an Eppendorf tube. When allantochorionic tissue was unavailable (*n* = 5), lung tissue was examined instead. The microtome blade was changed between each case.

DNA extraction was performed (QIAamp DNA FFPE Tissue Kit; Qiagen, Hilden, Germany) following the manufacturer's instructions and as described previously (19). Extracted DNA was examined (Nanodrop-1000; Witec, Lucerne, Switzerland) to determine DNA quantity and quality. Extracted DNA samples containing >120 ng/μL were diluted 1:10 in molecular biology grade water prior to real-time PCR to prevent amplification inhibition due to high DNA concentration.

All DNA samples were screened for the presence of *Chlamydiaceae* DNA using a real-time PCR assay targeting the 23S rRNA gene (*Chlamydiaceae* family-specific) (20) including primers Ch23S-F, Ch23S-R, and probe Ch23S-p (Microsynth, Balgach, Switzerland), as described previously (21). The internal amplification control eGFP amplified with primers eGFP-1-F, eGFP-10-R, and probe eGFP-Hex (Microsynth) (19) was added to each reaction. The PCR was conducted on a Thermocycler 7500-Fast ABI (ThermoFisher Scientific, Waltham, MA, USA). All samples were tested in duplicate, and samples with a cycle threshold of <38 from both PCR reactions were considered positive.

Real-time PCR-positive samples were further investigated using the Arraymate Microarray for chlamydial species identification, as described previously (22).

### Immunohistochemistry

Immunohistochemistry (IHC) for EHV-1 was performed on sections of lung, liver, spleen, adrenal gland, and allantochorion from cases that were PCR positive for EHV-1. Rabbit polyclonal antibodies against EHV-1 glycoprotein 2 (GP2; C-terminal) (ABIN966048; Antibodies-online.com) were used as the primary antibody and labeled with the UltraVision^TM^ ONE HRP polymer detection system (Thermo Scientific TL-125-HLJ).

Sections of 2 μm from the formalin-fixed paraffin-embedded tissue and a positive control (lung tissue from a PCR-confirmed EHV-1-infected equine fetus with typical lesions) were mounted on SuperFrost Plus™ Adhesion slides (Thermo Scientific), heated to 60°C for 30 min, deparaffinized in tissue clear, rehydrated in graded ethanol solutions and rinsed in running tap water. Antigen demasking was then performed by boiling in tris-EDTA buffer (pH 9.0) for 2 ×5 min in a microwave oven, followed by resting in the buffer for 15 min at room temperature. The slides were mounted on Shandon™ Sequenza™ slide racks (Thermo Scientific) and rinsed three times in Tris-buffered saline (TBS; Dako TBS S3001) (pH 7.6) for a total of 10 min. Endogenous peroxidases were inactivated with 3% H_2_O_2_ in TBS for 10 min at room temperature, followed by a wash in TBS as above. Potential non-specific binding sites were blocked to prevent non-specific antibody binding by applying a protein block (Thermo Scientific TA-125-PBQ) for 10 min followed by a wash in TBS as above. The sections were incubated with the primary antibody diluted 1:500 in antibody diluent (Agilent S0809) overnight at 4°C. A section incubated with normal rabbit serum (Agilent 0902) as a non-sense control was included. The following day, racks were acclimatized to room temperature for 15 min, rinsed in TBS as above, incubated with UltraVision^TM^ ONE HRP polymer for 30 min, rinsed again with TBS as above, and then incubated for 10 min in 3-amino-9-ethylcarbazole (AEC, Thermo Scientific TA-125-SA) to visualize binding sites. The sections were then washed twice in distilled water (10 min), removed from the slide racks, and counter-stained with Mayer's hematoxylin (10 s). Finally, the slides were rinsed under running tap water (1 min), rinsed in distilled water (4 min), and mounted with Kaiser's glycerol-gelatin.

### Diagnostic Criteria

The presence and severity of pathology was used as the main criterion for establishing a morphological diagnosis of the cause of pregnancy loss. Severity was qualitatively determined based on the type, distribution, and extent of lesions. Subsequent etiological diagnosis was based on the detection of an agent in a tissue with corresponding lesions.

In cases where several pathologies were identified, the presence and severity of each condition was assessed to determine which was the actual cause of fetal death. For example, in a fetus with severe umbilical cord torsion (UCT) characterized by segments of edema and hemorrhage separated by pale torsion furrows and concomitant alphaherpesvirus infection without associated lesions, the UCT was considered to be the cause of fetal death. However, if detected by PCR, EHV-1 and EAV were considered to be the cause of abortion, even in cases without characteristic pathology, unless a more obvious cause of fetal death was diagnosed. This approach was taken as these viruses may cause abortion due to the development of vascular lesions in the uterus, which could not be assessed in this study. In contrast, abortion due to EHV-4 should be accompanied by fetal or allantochorionic lesions, as the abortogenic potential of this virus is considered controversial.

## Results

**Figure 1:**
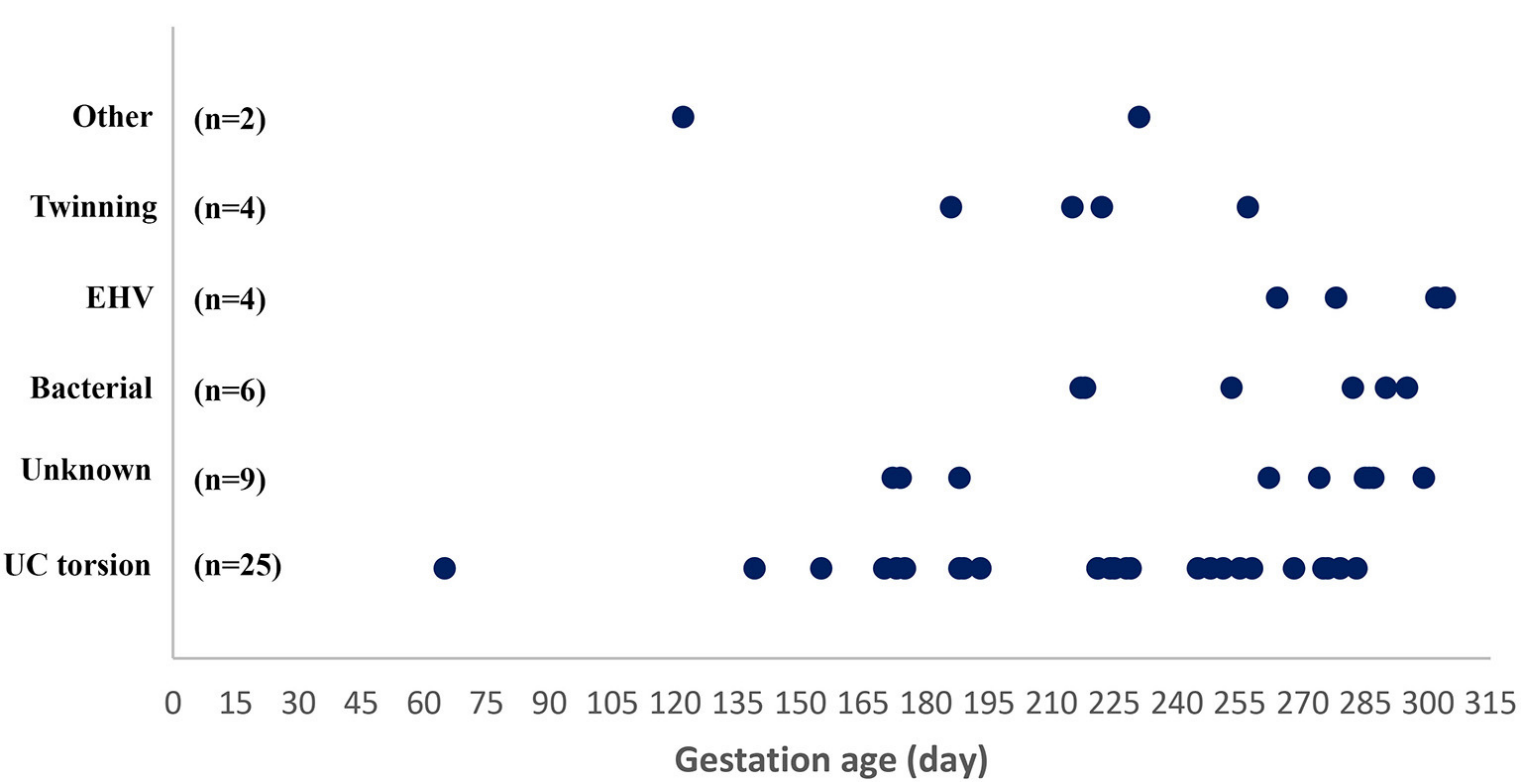
Gestational age at abortion or premature stillbirth in 50 mares grouped according to cause of pregnancy loss. UC torsion, umbilical cord torsion or other cord anomaly; EHV-1, equid alphaherpesvirus type 1.

Forty-five fetuses with corresponding fetal membranes and five fetuses without fetal membranes were submitted and deemed to be eligible for examination. These 50 cases consisted of 48 abortion cases and two premature stillborn foals. The main reason for not submitting the fetal membranes was their retention in the mare. Warmblood mares were most frequently represented (*n* = 30), followed by Icelandic horses (*n* = 7), ponies (*n* = 7), Standardbreds (*n* = 3), and other breeds (*n* = 3). The fetal gender ratio was 1:1. The fetuses were produced by insemination (30 cases), natural cover (18 cases), or embryo transfer (two cases). The gestational age at fetal expulsion ranged from 65 to 304 days, with the majority as abortion cases (*n* = 48). Abortion cases were mainly represented by fetuses aborted after GD 170 (44/48 cases) (Figure 1). Submissions came from most areas of Denmark (Supplementary Figure 1). The fetuses originated from 49 herds; only one herd submitted two cases. Thirteen mares (26.0%) were vaccinated against EHV-1 and EHV-4 during the gestational period. Lesions considered to be the most likely cause of fetal death were found in 41 cases (82.0%), with a causal diagnosis identified in 39/48 abortion cases and in 2/2 premature stillborn foals. In the following sections, abortions and premature stillbirths are reported separately.

### Abortion Cases

#### Umbilical Cord Lesions

Umbilical cord torsion was the most prevalent cause of abortion (25 cases; 52.1%) (Table 1), occurring in all breeds and throughout the fetal period (Figure 1). The youngest fetus with umbilical cord torsion was 65 days old and the oldest was 283 days. The cord length varied from 51 to 175 cm, the number of torsions ranged from 2 × 360° to 13 × 360° and involved both the intra-amniotic and intra-allantoic portions of the umbilical cord. Pathological changes other than the torsion *per se* (e.g., edema and congestion) were predominantly present in the intraamniotic portion, which was usually significantly longer than the intra-allantoic portion.

**Table 1:** Cause of abortion or premature stillbirth in 50 Danish mares.

**Cause**	**No**.	**Prevalence (%)**	**Comments**
Umbilical cord torsion or other cord anomalies	25	50	Concomitant latent infection with EHV-1 type N752 (*n* = 2), EHV-1 D752 (*n* = 1), EHV-4 (*n* = 1), or *Chlamydiaceae* sp. (*n* = 2). One case with scoliosis. One case of twinning.
Bacterial infection	6	12	Concomitant latent infection with EHV-4 (*n* = 1).
EHV-1	4	8	Type N752 (*n* = 2); D752 (*n* = 2).
Twinning	4	8	
Placental necrosis	1	2	
Fetal malformation	1	2	
Not determined	9	18	Abortion following diarrhea in the mare (*n* = 1). Infection with EHV-4 (*n* = 2).

*EHV-1, Equid alphaherpesvirus type 1; EHV-4, Equid alphaherpesvirus type 4*.

**Figure 2:**
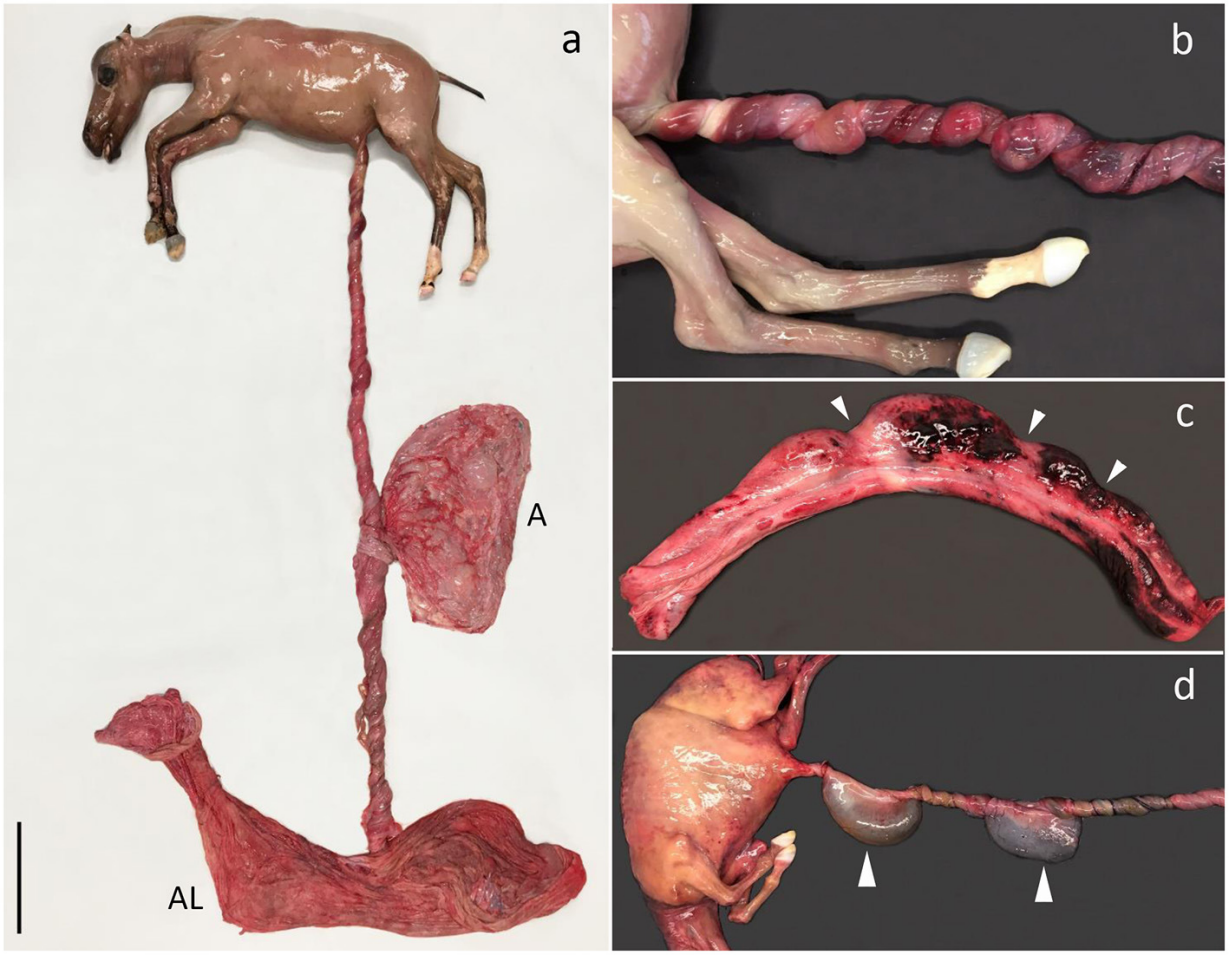
Umbilical cord torsion. **(a)** Fetus aborted due to multiple torsions of the umbilical cord (UC) (13 × 360°) involving both the intraamniotic and intra-allantoic portions. The UC had a total length of 175 cm. Aborted at gestation day (AGD) 224, A, amnion; AL, allantochorion. **(b)** Multiple torsions of the intraamniotic portion of the UC, AGD 221. **(c)** UC segment after untwisting of torsions. Notice the pale torsion furrows (arrowheads) separating distended segments of the UC with hemorrhage, AGD 243; **(d)** Multiple torsions of the intraamniotic portion of the UC. The torsions have obstructed the urinary flow through the allantoic duct and saccular ectasia of the duct has developed as a result (arrowheads). The size of the abdomen is increased due to urinary stasis causing extensive distension of the urinary bladder. AGD 155. All shown fetuses are Warmbloods.

Gross pathology was characterized by multiple torsions with pale furrows separating segments of the umbilical cord with edema, congestion, and hemorrhage (Figures 2a–c). The lumen of the umbilical arteries, vein, and allantoic duct varied in size depending on location in relation to the torsion furrows (i.e., the structures but especially the vein and duct were dilated between torsion furrows, and the vein was engorged with blood and compressed at the torsion sites. The urine flow had been severely obstructed in one case, causing significant dilation of the allantoic duct (Figure 2d), a severely distended urinary bladder, and bilateral hydronephrosis. Allantochorion was generally unremarkable, although a slight edema was present in some cases. UCT without segments of the umbilical cord with edema, congestion, and hemorrhage separated by torsion furrows were not considered pathologic and therefore not the cause of fetal death.

The fetuses had moderate-to-severe postmortem decomposition. Some fetuses had multifocal subpleural hemorrhage, but in general, the fetuses were macroscopically unremarkable. A concomitant thoracic scoliosis was present in one case and a mummified twin fetus was identified in another (weight 52 g vs. 5.7 kg in the twin with UCT).

Acute multifocal hemorrhage, congestion, and edema in the gelatinous substance of the umbilical cord (Wharton's jelly) and the amniotic lining were determined histologically. The appearance of the allantochorion varied from being unremarkable to cases with endothelial mineralization of vessels, sometimes with occlusion of the lumen, edema, and multifocal hemorrhage.

PCR revealed that three cases were simultaneously infected with EHV-1, one case with EHV-4, and two with *Chlamydiaceae* sp. None of these had microscopic lesions consistent with either EHV or *Chlamydiaceae* sp. infection. The two cases positive for *Chlamydiaceae* sp. were aborted at GD 245 and 268 with Ct values of 35 and 34, respectively—i.e., with a relatively small amount of bacterial DNA. It was not possible to identify the chlamydial species in these cases using Arraymate Microarray.

#### Bacterial Infections

Bacterial infection was diagnosed as the cause of abortion in six cases (12.5%). The age of the fetuses varied from GD 217 to GD 315 (Figure 1).

**Figure 3:**
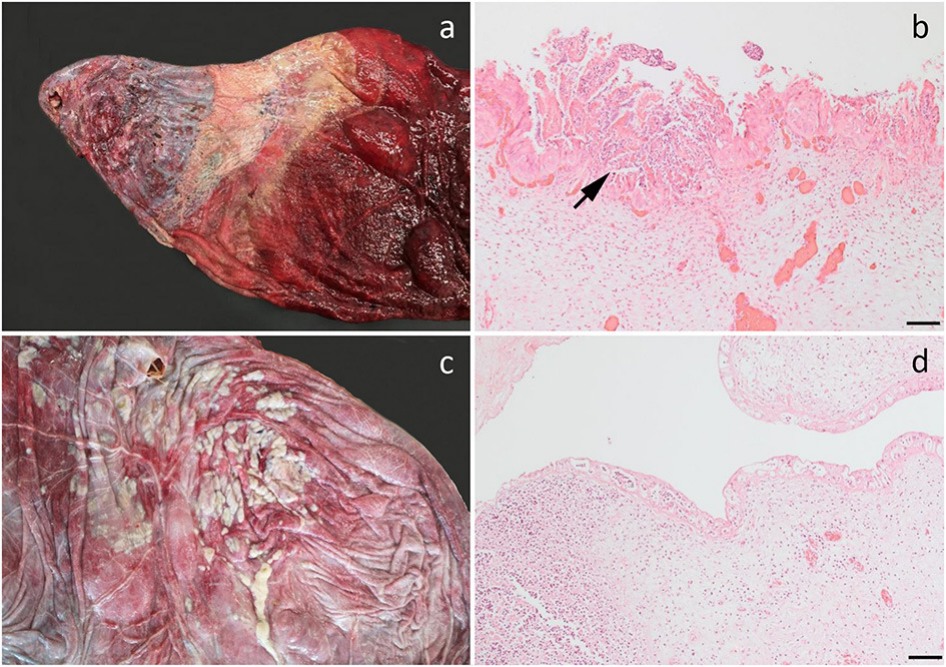
Bacterial infection of the fetal membranes. **(a)** Chronic necrosuppurative and fibrotic inflammation localized at the cervical pole of the chorion of a fetus aborted at gestation day (AGD) 253. Closest to the cervix, the allantochorion is fibrotic and is bordered by a zone of suppurative inflammation. The inflamed chorion has a rather distinct demarcation toward the grossly normal-appearing chorion (to the right). *Pseudomonas fluorescens* and *Arthrobacter gandavensis* were cultured from the lesion; **(b)** Photomicrograph of **(a)** showing a suppurative chorionitis (arrow); **(c)** Suppurative amnionitis in a fetus, AGD 217. *Enterococcus casseliflavus* was cultured in monoculture from the chorion, lung, liver, and stomach content; **(d)** Photomicrograph of **(c)** showing severe suppurative inflammation in the umbilical cord. **(b,d)** Hematoxylin and eosin, bar, 100 μm.

In one case, the allantochorion adjacent to the internal opening of the cervical canal had a circular pale thickened area representing fibrosis and loss of normal chorionic villous structures. This was bordered by a broad zone of yellowish discoloration of the allantochorion. Dual allantochorionic infection with *Pseudomonas fluorescens* and *Arthrobacter gandavensis* was diagnosed and histopathologically, the lesion was diagnosed as localized chronic necrosuppurative and fibrotic allantochorionitis (Figures 3a,b). Similarly, in another case, localized necrosuppurative allantochorionitis near the internal cervical opening was associated with isolation of *Acinetobacter hydrophila* in monoculture. Gross lesions were characterized by extensive fibrotic thickening of the allantochorionic body. The chorionic surface appeared pale and smooth without villous structures and the cervical star was difficult to identify due to fibrotic thickening in the area. The allantochorion was not ruptured at the cervical star area, but a chronic rupture of the body portion of the allantochorion with margins consisting of granulation tissue had developed and was associated with a dissecting separation of the feto-maternal-placental unit. The fetus was also positive for EHV-4, but neither microscopic nor macroscopic lesions indicative of viral infection were found.

*Staphylococcus vitulinus* was isolated in almost monoculture from the liver from a fetus with an enlarged liver with disseminated, locally coalescing pale foci. Histologically these represented an acute disseminated suppurative hepatitis. Fetal membranes were not submitted for this case. In another case, the umbilical cord of a 217-days old fetus was covered by numerous small abscesses, while the amniotic surface was partly covered by yellowish pus. Bacterial culture revealed a disseminated infection with *Enterococcus casseliflavus* in monoculture. Histopathologically, suppurative amnionitis, peripheral funisitis, profound suppurative inflammation of the allantois with vasculitis, thrombosis, and intravascular bacteria, suppurative hepatitis, and bronchopneumonia was diagnosed (Figures 3c,d).

In two cases, gross lesions were not found, but inflammation consistent with a bacterial etiology was found by histology. Subsequently, microbial culturing was made from frozen tissues. Fetal septicemia due to *Streptococcus equi* subsp. *zooepidemicus* was diagnosed in one of these cases. Inflammation was limited to a few groups of neutrophils and necrotic trophoblasts in the chorion, but numerous intravascular bacteria were present in multiple organs. Another case was diagnosed with suppurative allantochorionitis, bronchio-alveolitis, and interstitial myocarditis, but culturing failed to reveal an etiology. *Leptospira* spp. DNA was not detected in any cases.

#### Viral Infections

**Figure 4:**
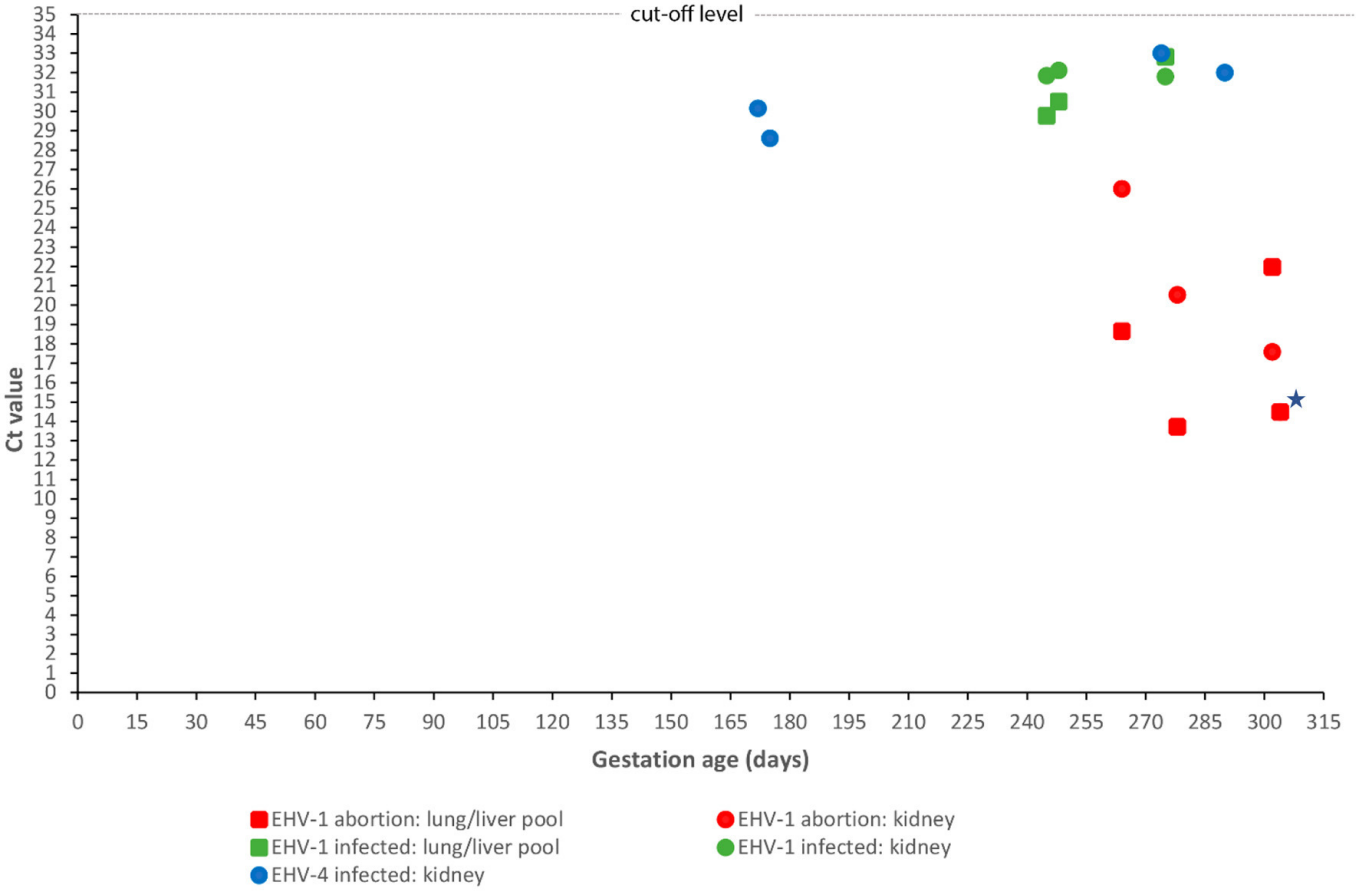
Correlation between fetal age at abortion or premature stillbirth and fetal viral load. Gestational age at abortion or premature stillbirth for 12 equine fetuses infected with either equid alphaherpesvirus type 1 (EHV-1) (*n* = 8) or type 4 (EHV-4) (*n* = 4) and Ct values obtained by real-time polymerase chain reaction of a kidney sample or a pool of lung and liver samples. Ct values for the pooled lung/liver samples for EHV-4 are not shown as all samples were negative, i.e., had a Ct value above 35. EHV-1 results have been divided into two groups: cases aborted due to EHV-1 and cases infected with EHV-1 but aborted due to another cause. A single case had equal Ct values in the kidney and pooled lung/liver samples (indicated by a star).

EHV-1 was the cause of abortion in two cases (4.0%); one was infected with variant N752 and the other with D752. The virus was found in all tissues analyzed, i.e., pooled lung/liver and kidney samples. The Ct value was lower than 27 for all cases/tissues and was mostly below 23 (Figure 4). Fetuses were aborted from GD 264 (Figure 1).

Necropsy revealed subcutaneous edema, abundant volumes of serosanguinous fluid in the thoracic cavity, disseminated hemorrhagic foci in the lung, and multifocal pale foci of ~2 mm in the liver. Fibrino-necrotizing and hemorrhagic broncho-interstitial pneumonia, acute to subacute necrosis in the liver and adrenal glands, necrosis of lymphoid follicles in the spleen, and eosinophilic intranuclear inclusion bodies were detected by histology. IHC demonstrated high levels of EHV-1 antigen in lung tissue.

The presence of EHV-1 DNA was found in another three cases, but without corresponding microscopic lesions. These cases all had Ct values above 29 (Figure 4). EHV-1 antigen was detected by IHC in trophoblasts in one case. Infection was caused by the variants N752 (*n* = 2) and D752 (*n* = 1). Umbilical cord torsion was identified as the cause of fetal death in all three cases. Two out of five mares that aborted fetuses infected with EHV-1 had been vaccinated with an inactivated EHV-1 vaccine but had received the last of the recommended three doses shortly before abortion.

Four cases tested positive for EHV-4 (8.0%) but none of these had lesions consistent with an EHV- like infection. The Ct values for kidney samples were above 28 for all cases, while all pooled lung/liver samples tested negative (Figure 4). The fetuses were aborted between GD 172 and 290 (Figure 4). Another likely cause of fetal death was identified for two of the cases. EAV DNA was not detected in any cases and none of the 50 mares had clinical signs indicating equine viral arteritis.

#### Twinning

Abortion due to twin pregnancy was diagnosed in four cases (Table 1) aborted between GD 186 and 257 (Figure 1). Both fetuses were submitted in two of these cases, while only one fetus was submitted for the other two cases, where abortion had occurred in the field. In the latter two cases, a presumptive diagnosis of twins was based on the presence of two sets of fetal membranes with an avillous chorionic portion.

#### Other Causes

**Figure 5:**
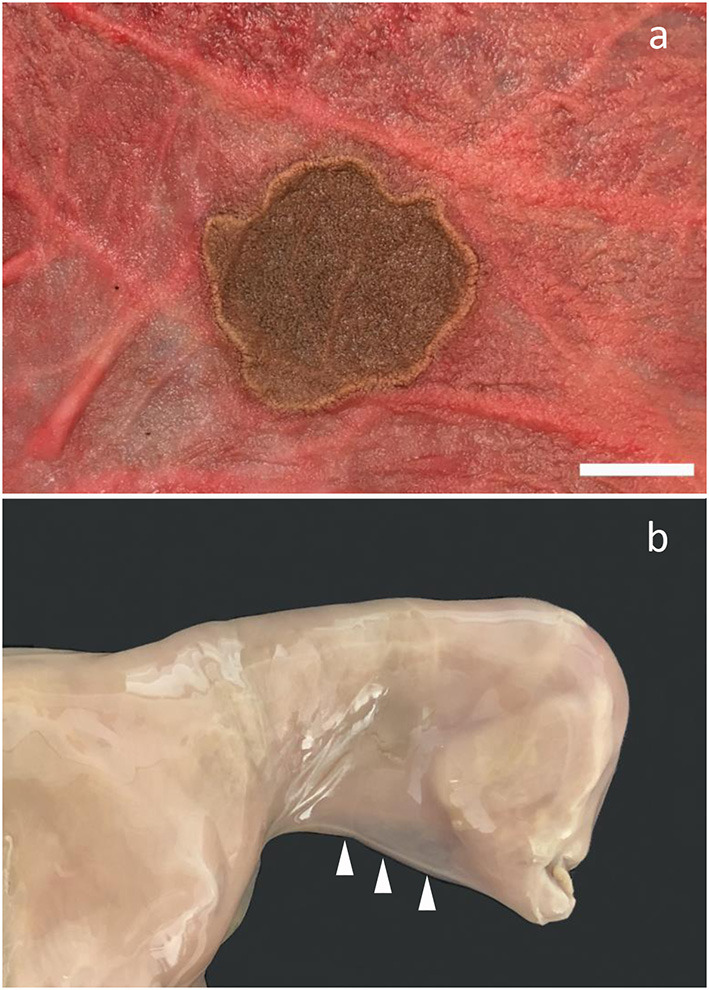
Other sporadic conditions found. **(a)** Localized necrosis in the allantochorion. Fetus aborted at gestation day 231. Bar = 2.5 cm; **(b)** Fetus aborted at gestation day 122. Notice anophthalmia, absence of pinna, superior brachygnathism, and the presence of a connective tissue band at the ventral midline of the head and neck (arrowheads).

Multiple circular round transmural necroses with diameters of up to 7 cm were present in the body of the allantochorion of a 231-day-old fetus (Figure 5a). The necrotic areas showed coagulation necrosis with calcification but with minimal cellular reaction. No pathogens were detected. The lesions were considered to be infarcts of unknown etiology. A congenital syndrome was diagnosed in a 122-day-old fetus with bilateral anophthalmia, absence of pinnae, superior brachygnathism, and the presence of a connective tissue band at the ventral midline of the head and neck (Figure 5b).

#### Cause Not Found

In nine cases (18.8%), no cause of abortion was identified. These abortions occurred during the second half of the gestation period (Figure 1). None of the mares showed signs of disease prior to spontaneously delivering a dead fetus, with the exception of one mare that developed persistent and progressive diarrhea 2 months before she aborted at GD 287.

### Premature Stillbirth

Both cases were premature stillbirths delivered at GD 302 and 304, respectively. At necropsy, one case had disseminated pale pin-point foci in the liver, while the other foal was inconspicuous although with low amounts of body fat. PCR analysis revealed that both fetuses were infected with EHV-1, with one case infected with variant N752 and the other with D752. The Ct values were below 22 for both cases. The N752 case had IHC-positive disseminated acute liver necroses as the main lesion, while the D752 case had lesions restricted to the allantochorion, although the liver, lung, and kidney were found to be positive by PCR. The allantochorionic lesions were characterized by extensive necrosis and loss of trophoblasts, calcification, stromal edema, and non-suppurative inflammation. EHV-1 antigens were present in the trophoblast layer, allantoic epithelium, and in the spleen. None of the mares had been vaccinated against EHV-1.

## Discussion

Torsion of the umbilical cord was diagnosed as the most prevalent cause of abortion in this Danish population of mares of various breeds. In general, the prevalence of abortion due to UCT and other umbilical cord anomalies varies between studies. In UK Thoroughbred populations, UCT has been identified as the most important cause of abortion, stillbirth, and neonatal death, with a prevalence of up to 45% (2, 5). Umbilical cord torsion alone accounted for 34.8% of abortion cases in the study by Roach et al. (2) (Roach, personal communication), but has been found to be less significant in other regions/studies, e.g., Germany (0.8%; 3.0%) (6, 11), Hungary (2.0%) (7), Michigan, USA (2.4%) (4), Kentucky, USA (3.4%) (1), Italy (5.8%) (9), and Japan (19%) (10). While some of the variation may be explained by differences in study design, availability of the umbilical cord at necropsy, different groupings and diagnostic criteria, and the high prevalence of certain abortifacients, etc., it is striking how prevalent UCT as cause of fetal loss appears to be in Thoroughbreds in the UK and in the heterogenous population of Danish mares reported here.

Bacterial infections are well-known causes of abortion in mares. Streptococci are considered to be major abortifacients and usually invade the uterus as an ascending infection through the cervix into the cervical pole of the allantochorion, and are associated with an initial development of localized allantochorionitis (23). *Streptococcus equi* subsp. *zooepidemicus* is commonly isolated from such cases (1, 4, 9, 11). However, in the present study, this species only accounted for one of six cases aborted due to bacterial infection.

Bacterial species not previously associated with equine abortion were identified in four cases. The bacteria, which included *E. casseliflavus, S. vitulinus, P. fluorescens, A. gandavensis*, and *A. hydrophila*, were isolated in almost monoculture—in some cases from several organs, and inflammation was evident by both gross and microscopic pathology (Figure 3). *E. casseliflavus* has previously been isolated from a foal with septic meningitis (24), *S. vitulinus* from bacterial extraocular disease, e.g., conjunctivitis (25), and *A. gandavensis* has been isolated from cases of equine amnionitis and fetal loss syndrome (26). The bacteria should be considered opportunistic pathogens that may cause abortion in mares if they reach the pregnant uterus. The case infected with *E. casseliflavus* had suppurative amnionitis, peripheral funisitis, and profound suppurative inflammation of the allantois. In addition, suppurative hepatitis and bronchopneumonia had also developed. The pathology resembled equine amnionitis and fetal loss syndrome (EAFLS) (8, 26), a condition not previously reported in Scandinavia. Both EAFLS and the related mare reproductive loss syndrome (27) are caused by maternal ingestion of processionary caterpillars (*Ochrogaster lunifer*), eastern tent caterpillars (*Malacosoma americanum*), or their exoskeletons (27, 28). These species are not found in Denmark, unlike the eastern pine processionary caterpillar (*Thaumetopoea pinivora*) and the oak processionary caterpillar (*Thaumetopoea processionea*). However, these live only in southern Denmark (29), while the mare lived in the most northern part of the country (Supplementary Figure 1). Furthermore, horses in Denmark do not usually graze in the habitats where these caterpillars live. Setae were not observed histologically, but they are rarely found in fetal membranes after day 22 post-exposure (28), so a lack of setae does not exclude an association with caterpillars. However, the pathogenesis of this case of amnionitis remains unsolved and its possible relation to caterpillar sp. is currently hypothetical.

Leptospirosis, which is a frequent cause of abortion in mares in some regions (30), was not diagnosed. The failure to detect leptospirosis cases may be due to the limited sample size (*n* = 50) or the fact that the PCR did not detect all *Leptospira* sp. However, leptospirosis in other domestic animal species is rare in Denmark, and although that does not exclude the presence of leptospirosis in horses, it may indicate that the rural environment in Denmark is not favorable to *Leptospira* sp. Bovine abortion due to leptospirosis has not been diagnosed (31, 32), and porcine leptospirosis has been limited to few herd outbreaks in sows kept on deep litter (33).

Equine abortions due to *Chlamydiaceae* involve the chlamydial species *Chlamydia abortus* and *C. psittaci* (34), with the latter also causing zoonotic infections in Australia. This study was unsuccessful in determining the chlamydial species in the two *Chlamydiaceae* PCR-positive cases. This might be because formalin fixation and putrefaction can cause DNA degradation. Moreover, the high Ct values in these two cases indicate a low DNA copy load, which, in connection with missing histological lesions, calls into question the importance of *Chlamydiaceae* as abortogenic agents. A recent retrospective study in Switzerland (35) detected *Chlamydiaceae* in only two equine abortion cases, both as co-infections with EHV-1, thus indicating that this pathogen might be of limited significance in equine abortion, at least in Switzerland and Denmark.

Abortion due to EHV-1 was diagnosed in 8% of the submissions, represented by two abortion cases and two premature stillbirths. All cases had lesions consistent with EHV-1 infection and the presence of EHV-1 was confirmed by PCR and IHC. EHV-1 is a well-known cause of abortion in mares (4–13). Viral DNA was also found in three abortion cases, which all had lesions consistent with fetal death due to UCT and lacked EHV-1 pathology. As a result, these were not considered to be primary EHV-1 abortion cases according to the applied diagnostic criteria, and the fetuses were considered to be in the incubation period.

Challenging late-term mares intranasally with a virulent strain of EHV-1 provoked abortion after 9–65 days (36–39), but a field study found that the period from the estimated date of exposure to abortion was up to 4 months (40). During leukocyte-associated viremia following exposure, the virus reaches the placenta 9–14 days after inoculation (36, 38, 41), after which the virus spreads to the fetus. Some fetuses will harbor the virus for a long period until the mare aborts or delivers an infected foal close to or at term. During the fetal incubation period, fetuses are at risk of developing other concomitant pathologies, and EHV-1-infected fetuses with other pathologies will therefore occur, as identified in the present study. Similar correlations between EHV-1 infection and other pathologies, including UCT, have been reported previously (41–43). In addition to abortion due to external exposure of the pregnant mare to a virulent strain, fetal infection may also result from the activation of a latent infection in the mare (44). The course of transmission of virus to the fetus and progression of fetal disease in such re-activations is poorly understood but may differ from experimental infections.

In the present study, two mares had completed a vaccination program but still aborted due to EHV-1. It has previously been reported that mares vaccinated against EHV-1 have aborted following exposure to an EHV-1 strain circulating in the herd (40, 45). In accordance with these observations, challenge studies of pregnant mares have shown that vaccination does not completely protect against abortion (37, 46). None of the EHV-1 abortion cases reported here originated from herds with recognized EHV-1 outbreaks, as clinical signs indicating a respiratory infection were not observed and only one horse per group aborted. The abortion cases may therefore represent the activation of a latent infection in these mares rather than a newly acquired infection.

Abortion due to maternal infection with EHV-1 is challenging to diagnose when based on examination of fetal organs alone. Experimental studies have revealed that some fetuses, particularly those aborting shortly after maternal exposure (i.e., 9–14 days after inoculation), may be virologically negative or that viral presence is restricted to the chorion or parts of it (36, 38, 41). Abortion in such cases is hypothesized to be due to virus-induced endometrial vasculitis accompanied by thrombosis leading to endometrial ischemia and consequently separation of the feto-maternal unit and fetal anoxia (41). The 8% prevalence found in the present study may therefore be an underestimate as the allantochorion was not subjected to PCR. As this is only relevant for acute infections, it is considered to be of limited significance, especially since no mares had a history of pyrexia or ocular or nasal discharge prior to the time of abortion/premature delivery. However, diagnosing EHV-1 abortion based on the presence of viral DNA without associated pathology in cases without another obvious cause of pregnancy loss may tend to overestimate the prevalence, as some fetuses may be aborted by an unrecognized cause during the EHV-1 incubation period. Associating EHV-1 with pregnancy loss is therefore challenging in some cases and emphasizes the need to define abortion criteria for use in equine abortion diagnostics. However, the use of real-time PCR targeting the *glycoprotein B* gene may prove to be useful. We observed that differentiation between EHV-1-associated abortion cases and EHV-1-infected fetuses aborted due to another cause was supported by the corresponding Ct values. EHV-1 abortion cases had a higher viral load than IHV-1-infected fetuses aborted due to another reason, mostly a Ct value <23 compared to >29 (Figure 4). These findings indicate that real-time PCR targeting the *glycoprotein B* gene may be useful when establishing the cause of abortion for fetuses infected with EHV-1. However, a larger sample of thoroughly investigated equine fetuses should be examined to evaluate the use of PCR testing for this purpose.

Infection with EHV-4 was diagnosed in four cases, two of which were aborted due to other recognized pathology and two without lesions (Table 1). The fetuses were apparently not viremic as only the kidney samples were found to be positive by PCR and with a low viral load (Ct values >28) in all cases (Figure 4). This level was similar to EHV-1-infected cases aborted due to another cause, but the EHV-1 cases were viremic, with the pooled lung/liver samples also testing positive. EHV-4 is a primary respiratory pathogen, but the virus may pass through the placenta and infect the fetus (47). However, EHV-4 is generally not considered a major cause of abortion (44). In this study, we applied strict diagnostic criteria based on the principle that the presence of agents should be reflected in lesions that could be considered abortogenic. This may underestimate some etiologies—including EHV-4. Our results confirm that EHV-4 can be present in fetuses without associated pathology or with only subtle lesions that are easily masked by autolysis. The consistent detection of EHV-4 DNA in the kidneys, i.e., a tissue that undergoes rapid autolysis, highlights the need for further investigation into the pathogenesis of fetal EHV-4 infection and the abortogenic capacity of the virus.

Infection with EAV was not identified, but similar to EHV-4, the diagnosis of EAV-associated abortion may be challenging. It is not always possible to detect virus and lesions in fetal tissues, as abortion may be due to uterine lesions such as myometrial vasculitis during the acute stage of the infection (48). None of the mares had any clinical signs indicative of EVA prior to abortion, but as abortion may follow even a subclinical infection (49), the absence of maternal illness does not completely rule out EAV as a cause of abortion.

Twinning is a well-known cause of fetal loss in mares, although the prevalence can be lowered by using ultrasound detection and embryo reduction (50). Furthermore, the twinning rate is breed dependent and comparison across studies is therefore challenging. Twinning is categorized as types A-C depending on how the fetuses share the endometrial capacity, as reflected by the size of the fetuses: dissimilar (type A), about equal size (B), or very dissimilar with one twin having undergone mummification (C) (51). All three types were observed in the present study.

Surveillance of animal populations for the causes of abortion is important for both animal health and financial reasons in the breeding society. This study was the first to investigate causes of abortion and premature stillbirth in mares in Denmark, and although a limited number of aborted fetuses and premature stillborn foals were examined, it provides an important insight into mare reproductive pathology in Denmark. Conditions such as UCT deserve more attention due to their significant role in pregnancy loss in mares, while it is also important to continue surveillance for pathogens and explore their abortogenic potential.

## Conclusions

This study is the first comprehensive investigation into the causes of abortion and premature stillbirth in mares in Denmark. As in the UK, but in contrast to several other regions, UCT was by far the most prevalent cause of abortion. This important influence of UCT highlights the need to investigate the reasons behind its geographically varying prevalence. Abortion due to bacterial infection accounted for 12% of cases. Among these, we identified an abortion case resembling EAFLS although the possible relation to caterpillars remains hypothetical. As this condition has not previously been reported in Scandinavia, veterinarians should be aware of this disease and submit materials for laboratory examination if amnionitis and funisitis are found. EHV-1 infection was associated with both abortion and premature stillbirth, as found in other studies. The results of the real-time PCR analysis for EHV-1 indicate that this method may be used to differentiate between abortions due to EHV-1 and fetuses infected with EHV-1 but aborted due to another cause. Furthermore, real-time PCR showed that EHV-4-infected fetuses were not viremic and had a low viral burden. The study was based on 50 submitted cases and was therefore limited in size, and other causes of abortion than those identified probably also exist in Denmark. It is advisable to continue surveillance of the Danish pregnant mare population to allow timely recognition of changes in patterns or the introduction of diseases new to Denmark.

## Data Availability Statement

The original contributions presented in the study are included in the article/Supplementary Material, further inquiries can be directed to the corresponding author/s.

## Ethics Statement

Ethical review and approval was not required for the animal study because the study not experimental and did not include live animals or animal euthanized for the study. The study was based on aborted and stillborn foals submitted for laboratory examination. Written informed consent was obtained from the owners for the participation of their animals in this study.

## Author Contributions

JSA conceived the study idea and design, performed the majority of necropsies, and drafted the manuscript. E-MK performed the PCR analysis for EHV-1 and 4, EVAV, and *Leptospira* sp. PD performed the culturing for bacteria and fungi. NB performed the analysis for *Chlamydiaceae* sp. HGP participated in the necropsy of abortion cases. MC participated in the necropsy of abortion cases and performed the IHC examination for EHV-1. All authors critically assessed the data, contributed intellectually to the manuscript, and read and approved the final version of the manuscript.

## Funding

This work was co-funded by the Horse Levy Foundation and supported by the contributing organizations.

## Conflict of Interest

E-MK is employed by Laboklin GmbH & Co., which is a company offering diagnostic tests. The remaining authors declare that the research was conducted in the absence of any commercial or financial relationships that could be construed as a potential conflict of interest.

## Publisher's Note

All claims expressed in this article are solely those of the authors and do not necessarily represent those of their affiliated organizations, or those of the publisher, the editors and the reviewers. Any product that may be evaluated in this article, or claim that may be made by its manufacturer, is not guaranteed or endorsed by the publisher.

## References

[1] Giles RC Donahue JM Hong CB Tuttle PA Petrites-Murphy MB Poonacha KB. Causes of abortion, stillbirth, and perinatal death in horses: 3,527 cases (1986–1991). J Am Vet Med Assoc. (1993) 203:1170–5. 8244867

[2] Roach JM Foote AK Smith KC Verheyen KL de Mestre AM. Incidence and causes of pregnancy loss after day 70 of gestation in Thoroughbreds. Equine Vet J. (2020) 2020:1–8. 10.1111/evj.13386 33205445

[3] Platt H. Aetiological aspects of abortion in the thoroughbred mare. J Comp Pathol. (1973) 83:199–205. 10.1016/0021-9975(73)90043-1 4796892

[4] Tengelsen LA Yamini B Mullaney TP Bell TG Render JA Patterson JS. A 12-year retrospective study of equine abortion in Michigan. J Vet Diagn Invest. (1997) 9:303–6. 10.1177/104063879700900312 9249170

[5] Smith KC Blunden AS Whitwell KE Dunn KA Wales AD. A survey of equine abortion, stillbirth and neonatal death in the UK from 1988 to 1997. Equine Vet J. (2003) 35:496–501. 10.2746/042516403775600578 12875329

[6] Hörügel U Pöhle D. Abortursachen bei Pferden in Sachsen von 2002-2007. Praktischer Tierarzt. (2008) 89:644–7.

[7] Szeredi L Tenk M Jánosi S Pálfi V Hotzel H Sachse K. A survey of equine abortion and perinatal foal losses in Hungary during a 3-year period (1998-2000). Acta Vet Hung. (2008) 56:353–67. 10.1556/avet.56.2008.3.9 18828487

[8] Todhunter KH Perkins NR Wylie RM Chicken C Blishen AJ Racklyeft DJ. Equine amnionitis and fetal loss: the case definition for an unrecognised cause of abortion in mares. Aust Vet J. (2009) 87:35–8. 10.1111/j.1751-0813.2008.00386.x 19178475

[9] Marenzoni ML Lepri E Casagrande Proietti P Bietta A Coletti M Timoney PJ Passamonti F. Causes of equine abortion, stillbirth and neonatal death in central Italy. Vet Rec. (2012) 170:262. 10.1136/vr.100551 22368162

[10] Murase H Miyazawa M Harada T Ozawa M Sato F Hada T. Aborted fetal sizes of thoroughbred horses in Hidaka, Japan, between 2005 and 2015. J Equine Sci. (2017) 28:47–53. 10.1294/jes.28.47 28721123 PMC5506449

[11] Weber R Hospes R Wehrend A. Abortursachen beim Pferd – eine Übersicht der Literatur und eigene Auswertungen. Tierarztl Prax Ausg G Grosstiere Nutztiere. (2018) 46:35–42. 10.15653/TPG-170517 29536469

[12] Akter R Legione A Sansom FM El-Hage CM Hartley CA Gilkerson JR. Detection of *Coxiella burnetii* and equine herpesvirus 1, but not *Leptospira* spp. or *Toxoplasma gondii*, in cases of equine abortion in Australia - a 25 year retrospective study. PLoS ONE. (2020) 15:e0233100. 10.1371/journal.pone.0233100 32453753 PMC7250447

[13] Silva AA Villalobos EMC Cunha EMS Lara MCCSH Nassar AFC Piatti RM. Causes of equine abortion, stillbirth, and perinatal mortality in Brazil. Arq Inst Biol. (2020) 87:1–9. 10.1590/1808-1657000092020

[14] Diallo IS Hewitson G Wright L Rodwell BJ Corney BG. Detection of equine herpesvirus type 1 using a real-time polymerase chain reaction. J Virol Methods. (2006) 13:92–8. 10.1016/j.jviromet.2005.07.010 16137772

[15] Diallo IS Hewitson G Wright LL Kelly MA Rodwell BJ Corney BG. Multiplex real-time PCR for the detection and differentiation of equid herpesvirus 1 (EHV-1) and equid herpesvirus 4 (EHV-4). Vet Microbiol. (2007) 123:93–103. 10.1016/j.vetmic.2007.02.004 17346907

[16] Nugent J Birch-Machin I Smith KC Mumford JA Swann Z Newton JR. Analysis of equid herpesvirus 1 strain variation reveals a point mutation of the DNA polymerase strongly associated with neuropathogenic disease outbreaks. J Virol. (2006) 80:4047–60. 10.1128/JVI.80.8.4047-4060.2006 16571821 PMC1440451

[17] Lu Z Branscum AJ Shuck KM Zhang J Dubovi EJ Timoney PJ. Comparison of two real-time reverse transcription polymerase chain reaction assays for detection of equine arteritis virus nucleic acid in equine semen and tissue culture fluid. J Vet Diagn Invest. (2008) 20:147–55. 10.1177/104063870802000202 18319426

[18] Stoddard RA Gee JE Wilkins PP McCaustland K Hoffmaster AR. Detection of pathogenic *Leptospira* spp. through TaqMan polymerase chain reaction targeting the LipL32 gene. Diagn Microbiol Infect Dis. (2009) 64:247–55. 10.1016/j.diagmicrobio.2009.03.014 19395218

[19] Borel N Marti H Pospischil A Pesch T Prähauser B Wunderlin S. *Chlamydiae* in human intestinal biopsy samples. Pathog Dis. (2018) 76:fty081. 10.1093/femspd/fty081 30445531 PMC6276272

[20] Everett KD Bush RM Andersen AA. Emended description of the order *Chlamydiales*, proposal of *Parachlamydiaceae* fam. nov. and *Simkaniaceae* fam. nov., each containing one monotypic genus, revised taxonomy of the family *Chlamydiaceae*, including a new genus and five new species, and standards for the identification of organisms. Int J Syst Bacteriol. (1999) 49:415–40. 10.1099/00207713-49-2-415 10319462

[21] Ehricht R Slickers P Goellner S Hotzel H Sachse K. Optimized DNA microarray assay allows detection and genotyping of single PCR-amplifiable target copies. Mol Cell Probes. (2006) 20:60–3. 10.1016/j.mcp.2005.09.003 16330186

[22] Borel N Kempf E Hotzel H Schubert E Torgerson P Slickers P. Direct identification of *Chlamydiae* from clinical samples using a DNA microarray assay: a validation study. Mol Cell Probes. (2008) 22:55–64. 10.1016/j.mcp.2007.06.003 17714911

[23] Prickett ME. Abortion and placental lesions in the mare. J Am Vet Med Assoc. (1970) 157:1465–70. 4922185

[24] Stefanetti V Beccati F Passamonti F Sgariglia E Coletti M Vuerich M. Detection and DNA quantification of *Enterococcus casseliflavus* in a foal with septic meningitis. J Am Vet Med Assoc. (2016) 249:96–100. 10.2460/javma.249.1.96 27308888

[25] Hidaka S Kobayashi M Ando K Fujii Y. Efficacy and safety of lomefloxacin on bacterial extraocular disease in the horse. J Vet Med Sci. (2015) 77:829–35. 10.1292/jvms.14-0507 25787926 PMC4527505

[26] Todhunter KH Muscatello G Blishen AJ Chicken C Perkins NR Gilkerson JR. Bacteria isolated from field cases of equine amnionitis and fetal loss. Aust Vet J. (2013) 91:138–142. 10.1111/avj.12022 23521098

[27] Sebastian MM Bernard WV Riddle TW Latimer CR Fitzgerald TD Harrison LR. Review paper: mare reproductive loss syndrome. Vet Pathol. (2008) 45:710–22. 10.1354/vp.45-5-710 18725479

[28] Todhunter KH Cawdell-Smith AJ Bryden WL Perkins NR Begg AP. Processionary caterpillar setae and equine fetal loss: 1. Histopathology of experimentally exposed pregnant mares. Vet Pathol. (2014) 51:1117–30. 10.1177/0300985813516639 24379221

[29] Fibiger M Top-Jensen M. Danske Sommerfugle. Østermarie: Bugbook Publishing (2009). p. 678.

[30] Donahue JM Williams NM. Emergent causes of placentitis and abortion. Vet Clin North Am Equine Pract. (2000) 16:443–56. 10.1016/S0749-0739(17)30088-3 11219342

[31] Agerholm JS Willadsen CM Nielsen TK Giese SB Holm E Jensen L. Diagnostic studies of abortion in Danish dairy herds. Zentralbl Veterinarmed A. (1997) 44:551–8. 10.1111/j.1439-0442.1997.tb01141.x 9465775

[32] Wolf-Jäckel GA Hansen MS Larsen G Holm E Agerholm JS Jensen TK. Diagnostic studies of abortion in Danish cattle 2015–2017. Acta Vet Scand. (2020) 62:1. 10.1186/s13028-019-0499-4 31900210 PMC6942357

[33] Friis NF Jorsal SE Sørensen V Schirmer AL Lindahl J Thorup F. Enzootics of *Leptospira* abortions in Danish sow herds practising loose housing on deep straw bedding. Acta Vet Scand. (2000) 41:387–90. 10.1186/BF03549629 11234972 PMC7996430

[34] Borel N Polkinghorne A Pospischil A. A review on chlamydial diseases in animals: still a challenge for pathologists? Vet Pathol. (2018) 55:374–90. 10.1177/0300985817751218 29310550

[35] Baumann S Gurtner C Marti H Borel N. Detection of Chlamydia species in 2 cases of equine abortion in Switzerland: a retrospective study from 2000 to 2018. J Vet Diagn Invest. (2020) 32:542–8. 10.1177/1040638720932906 32522107 PMC7438654

[36] Smith KC Whitwell KE Blunden AS Bestbier ME Scase TJ Geraghty RJ. Equine herpesvirus-1 abortion: atypical cases with lesions largely or wholly restricted to the placenta. Equine Vet J. (2004) 36:79–82. 10.2746/0425164044864732 14756377

[37] Heldens JG Hannant D Cullinane AA Prendergast MJ Mumford JA Nelly M. Clinical and virological evaluation of the efficacy of an inactivated EHV1 and EHV4 whole virus vaccine (Duvaxyn EHV1,4). Vaccination/challenge experiments in foals and pregnant mares. Vaccine. (2001) 19:4307–17. 10.1016/S0264-410X(01)00131-1 11457558

[38] Patel JR Bateman H Williams J Didlick S. Derivation and characterisation of a live equid herpes virus-1 (EHV-1) vaccine to protect against abortion and respiratory disease due to EHV-1. Vet Microbiol. (2003) 91:23–39. 10.1016/S0378-1135(02)00259-6 12441229

[39] Gardiner DW Lunn DP Goehring LS Chiang YW Cook C Osterrieder N. Strain impact on equine herpesvirus type 1 (EHV-1) abortion models: viral loads in fetal and placental tissues and foals. Vaccine. (2012) 30:6564–72. 10.1016/j.vaccine.2012.08.046 22944628

[40] Mumford JA Rossdale PD Jessett DM Gann SJ Ousey J Cook RF. Serological and virological investigations of an equid herpesvirus 1 (EHV-1) abortion storm on a stud farm in 1985. J Reprod Fertil Suppl. (1987) 35:509–18. 2824770

[41] Smith KC Whitwell KE Binns MM Dolby CA Hannant D Mumford JA. Abortion of virologically negative foetuses following experimental challenge of pregnant pony mares with equid herpesvirus 1. Equine Vet J. (1992) 24:256–9. 10.1111/j.2042-3306.1992.tb02830.x 1323457

[42] Ellis WA Bryson DG McFerran JB. Abortion associated with mixed *Leptospira*/equid herpesvirus 1 infection. Vet Rec. (1976) 98:218–9. 10.1136/vr.98.11.218 178087

[43] Dunn KA Smith KC Blunden AS Wood JL Jagger DW. EHV-1 infection in twin equine fetuses. Vet Rec. (1993) 133:580. 8303812

[44] Patel JR Heldens J. Equine herpesviruses 1 (EHV-1) and 4 (EHV-4) - epidemiology, disease and immunoprophylaxis: a brief review. Vet J. (2005) 170:14–23. 10.1016/j.tvjl.2004.04.018 15993786

[45] Damiani AM de Vries M Reimers G Winkler S Osterrieder N. A severe equine herpesvirus type 1 (EHV-1) abortion outbreak caused by a neuropathogenic strain at a breeding farm in northern Germany. Vet Microbiol. (2014) 172:555–62. 10.1016/j.vetmic.2014.06.023 25042527

[46] Bürki F Rossmanith W Nowotny N Pallan C Möstl K Lussy H. Viraemia and abortions are not prevented by two commercial equine herpesvirus-1 vaccines after experimental challenge of horses. Vet Q. (1990) 12:80–6. 10.1080/01652176.1990.9694249 2163560

[47] Van Maanen C. Equine herpesvirus 1 and 4 infections: an update. Vet Q. (2002) 24:58–78. 10.1080/01652176.2002.9695126 12095082

[48] Coignoul FL Cheville NF. Pathology of maternal genital tract, placenta, and fetus in equine viral arteritis. Vet Pathol. (1984) 21:333–40. 10.1177/030098588402100311 6328724

[49] Timoney PJ. Equine viral arteritis. In: McKinnon AO, Squire EL, Vaala WE, Varner DD, editors, *Equine Reproduction*. 2nd ed. vol. 2. Hoboken, NJ: Blackwell Publishing Ltd (2011). p. 2391–8.

[50] Macpherson ML Reimer JM Twin reduction in the mare: current options. Anim Reprod Sci. (2000) 60–1:233–44. 10.1016/S0378-4320(00)00112-3 10844198

[51] Jeffcott LB Whitwell KE. Twinning as a cause of foetal and neonatal loss in the thoroughbred mare. J Comp Pathol. (1973) 83:91–106. 10.1016/0021-9975(73)90032-7 4731313

