# Supplementary material for: A Diagnostic Survey of Aborted Equine Fetuses and Stillborn Premature Foals in Denmark
Source: Front Vet Sci. 2021 Nov 10;8:740621. doi: 10.3389/fvets.2021.740621 (PMC8631530; doi:10.3389/fvets.2021.740621)

Supplementary Figure 1. Map of Denmark indicating the location of herds from which aborted fetuses or premature stillborn foals were submitted for examination.

The 50 submissions originated from 49 herds covering most areas of Denmark. Only one herd submitted two cases, and both were positive for equid herpesvirus (EHV) type 4. The location of cases that tested positive for EHV type 1 are shown in red, those positive for EHV type 4 are shown in green, and the amnionitis case is shown in orange. The location of the Department of Veterinary Clinical Sciences is indicated by a star.

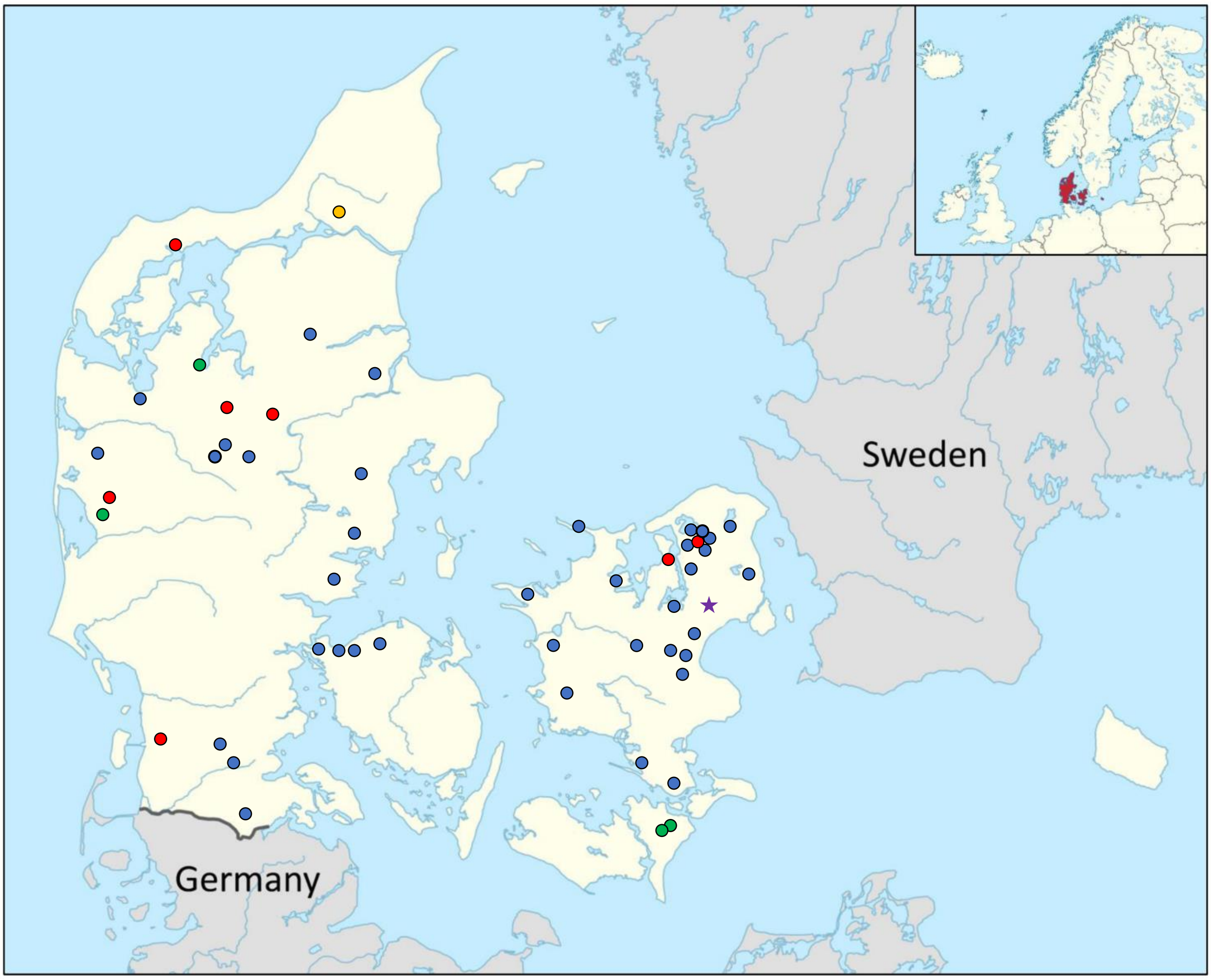

Supplement: Supplementary file 1 [file Data_Sheet_1.pdf]
