# Supplementary material for: A Diagnostic Survey of Aborted Equine Fetuses and Stillborn Premature Foals in Denmark
Source: Front Vet Sci. 2021 Nov 10;8:740621. doi: 10.3389/fvets.2021.740621 (PMC8631530; doi:10.3389/fvets.2021.740621)
Supplement: Supplementary file 2 [file Table_1.DOCX]

**Supplementary Table 1**. Overview of fetuses retrospectively excluded from the study due to not fulfilling the inclusion criteria

| **Exclusion Case no.** | **Gestational age (GA)** | **Breed** | **Diagnosis** | **Cause of exclusion** |
| --- | --- | --- | --- | --- |
| 1 | Unknown | Warmblood | EHV-1 | No data provided |
| 2 | 342 | Warmblood | Hepatitis^1^ | GA >315 days |
| 3 | 336 | Jutland horse | Pneumonia^2^ | GA >315 days |
| 4 | 270 | Warmblood | Umbilical cord torsion | Severely decomposed |
| 5 | 348 | Warmblood | Not established | GA >315 days |
| 6 | 322 | Warmblood | Not established | GA >315 days |
| 7 | 332 | Icelandic | Not established | GA >315 days |
| 8 | 331 | Standardbred | Not established | GA >315 days |
| 9 | 354 | Warmblood | Not established | GA >315 days |
| 10 | 306 | Knabstrup horse | Not established | Fetus frozen |
| 11 | 324 | Icelandic | Not established | GA >315 days |

EHV-1: Equine herpesvirus type 1 variant N752; ^1^ Acute suppurative hepatitis, 2-3 foci. Examined tissues sterile; ^2^ *Streptococcus equi* subsp. *zooepidemicus* and *Escherichia coli* were isolated from lung and liver, while an unspecific flora was cultured from the placenta.
